# Circ_0003998 enhances doxorubicin resistance in hepatocellular carcinoma by regulating miR-218-5p/EIF5A2 pathway

**DOI:** 10.1186/s13000-020-01056-1

**Published:** 2020-12-11

**Authors:** Xiaomin Li, Jiefeng He, Xiaojing Ren, Haichao Zhao, Haoliang Zhao

**Affiliations:** 1 Shanxi Medical University, Taiyuan, Shanxi China; 2 Department of General Surgery, Shanxi Bethune Hospital, No. 99 Longcheng Street, Xiaodian District, Taiyuan, 030032 Shanxi China

**Keywords:** circ_0003998, miR-218-5p, EIF5A2, HCC, Doxorubicin resistance

## Abstract

**Background:**

The involvement of circular RNAs (circRNAs) in chemoresistance of tumors has been identified. Herein, this study aims to investigate the role and the underlying mechanism of circ_0003998 in doxorubicin (DOX) resistance in hepatocellular carcinoma (HCC).

**Methods:**

The expression of circ_0003998 and microRNA (miR)-218-5p and eukaryotic translation initiation factor 5A-2 (EIF5A2) mRNA was detected using quantitative real-time polymerase chain reaction. Cell viability, migration and invasion were analyzed using cell counting kit-8, colony formation and transwell assay, respectively. The levels of matrix metallopeptidase 9 (MMP-9), E-cadherin, Vimentin, N-cadherin and EIF5A2 protein were detected using western blot. The interaction between miR-218-5p and circ_0003998 or EIF5A2 was confirmed by dual-luciferase reporter assay. In vivo experiments were performed using murine xenograft models.

**Results:**

Circ_0003998 was elevated in HCC tissues, DOX-resistant tissues and cells, and circ_0003998 knockdown promoted DOX-sensitivity in HCC by inhibiting resistant cell viability, migration, invasion and EMT in vitro and enhanced DOX cytotoxicity in vivo. Bioinformatics analysis revealed circ_0003998 inhibited miR-218-5p expression, which was clarified to be a target of circ_0003998, and circ_0003998 knockdown sensitized HCC cell to DOX by sponging miR-218-5p. EIF5A2 was a target of miR-218-5p, and miR-218-5p mitigated DOX resistance in HCC cells through modulating EIF5A2 expression. Additionally, circ_0003998 served as a competing endogenous RNA for miR-218-5p to regulate EIF5A2 expression.

**Conclusion:**

Circ_0003998 knockdown sensitized HCC cell to DOX by regulating miR-218-5p/EIF5A2 axis, indicating new markers of poor response to DOX and potential therapeutic strategies for the chemotherapy of HCC.

**Supplementary Information:**

The online version contains supplementary material available at 10.1186/s13000-020-01056-1.

## Highlight

1. Circ_0003998 and EIF5A2 are elevated, while miR-218-5p is decreased in HCC tissues and DOX-resistant HCC cell lines.
2. Circ_0003998 knockdown sensitizes HCC cell to DOX in vitro and promotes DOX cytotoxicity in vivo.
3. MiR-218-5p directly binds to circ_0003998 and EIF5A2.
4. Circ_0003998 can negatively regulate EIF5A2 by binding to miR-218-5p.
5. Circ_0003998 knockdown promotes DOX-sensitivity in HCC through miR-218-5p/EIF5A2 axis.

## Introduction

Hepatocellular carcinoma (HCC), as one of the most common global leading causes of cancer-related mortality, is an invasive malignant tumor and severely threaten human health [1]. Surgical resection is the most effective therapy for early-stage HCC, while chemotherapy may be the only method widely adopted to prolong survival in patients with intermediate and advanced HCC [2, 3]. Doxorubicin (DOX) is one kind of anthracycline-based chemotherapeutic agent that suppresses DNA/RNA synthesis trough intercalation between base pairs of DNA strands, thus leading to cancer cells apoptosis [4]. DOX is a first-line chemotherapy strategy for transarterial chemoembolization (TACE), a most commonly used treatment for most advanced HCC [5, 6]. However, DOX resistance gradually emerged in HCC patients to limit the effects of the drug [7]. Thus, better understanding on the mechanisms of DOX resistance in HCC is of great significance.

Circular RNAs (circRNAs) are endogenous, highly conserved small non-coding RNAs with a closed structure lacking the 5′-end cap and the 3′-end poly (A) tail, which make them more biologically stable than linear RNA [8]. Growing evidence have revealed that dysregulated circRNAs participate in the regulation of tumor cell carcinogenesis, progression and chemoresistance [9, 10]. Hsa_circ_0003998 is a circRNA with 304 nucleotides in spliced sequence length, which gene is mapped on chr20:47570092–47,580,435. It has been found the expression of hsa_circ_0003998 is abnormal in renal cell carcinoma and breast cancer [11], indicating hsa_circ_0003998 might be associated with the progression of cancers. Subsequently, Yu et al. discovered hsa_circ_0003998 interacted with microRNA (miR)-326 to accelerate cell proliferation and invasion in non-small cell lung cancer (NSCLC) [12]. Moreover, they also showed hsa_circ_0003998 knockdown sensitized lung adenocarcinoma cell to docetaxel by inhibiting proliferation and promoting apoptosis of resistant cells through miR-326 [13]. Besides that, a recent study suggested that circ_0003998 was elevated in HCC, and was associated with HCC diagnosis and prognosis [14], revealing the potential roles of hsa_circ_0003998 in HCC progression. Therefore, we assumed that circ_0003998 might regulate DOX resistance in HCC.

Here, this study aimed to explore the function of circ_0003998 in DOX resistance in HCC cells, and investigated the related target genes and molecular mechanism of circ_0003998 on DOX resistance.

## Materials and methods

### Clinical samples

From August 2017 to March 2019, a total of 55 paired primary HCC tissues and adjacent normal tissues who underwent hepatectomy were obtained from Shanxi Medical University. All included patients only underwent doxorubicin (DOX)-based neo-adjuvant chemotherapy (50-60 mg/mL) at least six cycles prior to surgery. Besides, patients suffered from other malignancies, received other combined chemotherapies and/or radiotherapy, or who had been or were being administered with other medications were excluded. The fresh samples were stored at − 80°Cuntil further experiments. Pathological examinations were performed in line with the Solid Tumor Response Evaluation Criteria (RECIST), of which 25 specimens were sensitive (CR + PR) and the rest were resistant (SD + PD, *N* = 30). This study was permitted by the Ethics Committee of Shanxi Medical University and written informed consent was collected from all patients.

### Quantitative real-time polymerase chain reaction (qRT-PCR)

The extraction of total RNA was carried out using TRIzol reagent (Invitrogen, Carlsbad, CA, USA) according to the standard procedure. For the detection of circRNA, extracted RNAs were treated with RNase R to digest linear RNA. Then complementary DNAs (cDNAs) were synthesized by using All-in-One™ Kit (GeneCopoeia, Rockville, MD, USA). Then synthesized cDNA template was supplemented with SYBR Green I (Takara, Dalian, China). The thermocycling conditions were as follows: Initial denaturation at 95 °C for 3 min; 40 cycles of 95 °C for 5 s and 60 °C for 30 s. A melt curve step from 65 to 95 °C was conducted in increments of 0.5 °C per 5 s. The relative expression was calculated by the 2^−ΔΔCt^ method using glyceraldehyde-3-phosphate dehydrogenase (GAPDH) and U6 small nuclear B noncoding RNA (U6) as normalization controls. The primers were listed as followed: circ_0003998: F 5′-CAGGAGGTGGTGAAGGACAT-3′, R 5′-CCTGACTGTGCTTCAAACGA-3′; miR-218-5p: F 5′-AACACGAACTAGATTGGTACA-3′, R 5′-AGTCTCAGGGTCCGAGGTATTC-3′. Eukaryotic translation initiation factor 5A-2 (EIF5A2), F 5′-GGACGACCATGCAAAATAGTGG-3′, and R 5′-TGCCCGTGAAAATATCAATTCCA-3′; GADPH: F 5′-CCCACATGGCCTCCAAGGAGTA-3′, R 5′-GTGTACATGGCAACTGTGAGGAGG-3′; U6: F 5′-CTCGCTTCGGCAGCACA-3′, R 5′- CGCTTCACGAATTTGCGTGTCAT-3′.

### RNase R digestion assay

Total RNA (10 μg) was incubated with 3 U/μg of RNase R (Epicentre Technologies, Madison, WI, USA) for 20 min at 37 °C. Total RNA untreated with RNase was used as a control. Then the levels of circ_0003998 and linear mRNA were determined using qRT-PCR assay.

### Cell culture

HCC cell lines (Hep3B, Huh7 and HCCLM3) and normal hepatocyte THLE-2 were purchased from Shanghai Academy of Life Science (Shanghai, China). All cells were grown in Dulbecco’s modified Eagle’s medium (DMEM, GIBCO, New York, NY, USA) harboring with 10% fetal bovine serum (FBS) and penicillin/streptomycin (100 U/mL, GIBCO) with 5% CO_2_ at 37 °C.

Doxorubicin-resistant Huh7 (Huh7/DOX) and HCCLM3 (HCCLM3/DOX) cells were generated by gradually exposing DOX-sensitive Huh7 and HCCLM3 cells to increasing doses of DOX (0.5–25 μg/mL, Sigma, San Francisco, CA, USA) over a 6-month period. DOX-resistant cells were cultured in the same media added with 0.1 μg/mL DOX to retain the DOX-resistant phenotype.

### Cell transfection

The mimic and inhibitor of miR-218-5p (miR-218-5p and anti-miR-218-5p) and their negative control (miR-NC and anti-miR-NC) were obtained from RIBOBIO (Guangzhou, China). Small interfering RNA (siRNA) targeting circ_0003998 covalent closed junction (si-circ_0003998) or siRNA negative control (si-NC), the scramble shRNA sequence or shRNA targeting circ_0003998 (sh-NC or sh-circ_0003998), pDNA-EIF5A2 overexpression vector (pcDNA-EIF5A2) and empty plasmid (pcDNA-NC) were synthesized by Invitrogen. Afterwards, DOX-resistant cells were plated in six-well plate (5 × 10^5^ cells/well), allowed to adhere for 24 h and transfected with these siRNC (10 nM), miRNA mimics or inhibitor (30 nM) or pcDNAs (10 nM) using Lipofectamine 2000 (Invitrogen).

### Cell viability assay

Transfected resistant cells were seed into 96-well plates (5000 cell/well) overnight, and then were treated with increasing concentrations of DOX (0, 0.0625, 0.125, 0.25, 0.5, or 1 μg/mL) for incubating for another 48 h. After that, per well was incubated with cell counting kit-8 (CCK-8) solution (10 μL/well) (Beyotime, Shanghai, China) for about 2 h. Subsequently, the optical density at 450 nm was determined by a microplate reader.

### Colony formation assay

Transfected resistant cells (500/well) were placed on 6-well plates with 0.5 μg/mL DOX and maintained for 21 days. Subsequently, cells were fixed with methanol and stained with 0.1% crystal violet (Sigma) and the visible colonies (≥50 cells) were counted and the typical images were photographed.

### Cell migration and invasion assay

Transwell chamber membranes were pre-coated without or with Matrigel (BD Biosciences, San Jose, CA, USA) to determine the abilities of migration and invasion of resistant cells, respectively. Following transfection, resistant cells suspended in serum-free DMEM were seed in the upper chamber of transwell, and 500 μL DMEM containing with 10% FBS was added into the lower chambers. After 48 h, cells on the lower face of the membranes were fixed and stained, and recorded by a microscope.

### Western blot

After transfection, resistant cells were lysed by RIPA lysis buffer (Beyotime) to isolate proteins, and then protein concentrations were analyzed by bicinchoninic acid (BCA) Protein Assay kit (Takara). Subsequently, equal amounts of proteins (30 μg) were separated by sodium dodecyl sulfate polyacrylamide gel electrophoresis, and shifted to polyvinylidene fluoride membranes. Afterwards, the membranes were interacted with primary antibody anti-matrix metallopeptidase 9 (MMP-9) (1:2000, ab38898), anti-E-cadherin (1:1000, ab15148), anti-Vimentin (1:5000, ab92547), anti-N-cadherin (1:3000, ab18203) anti-EIF5A2 (1:5000; ab150439) (Abcam, Cambridge, MA, USA) and the secondary antibody anti-rabbit IgG-horseradish peroxidase (1:1000, Sangon, Shanghai, China). GADPH (1:10000, ab8245, Abcam) was used as a normalization control. Finally, immunoreactive bands were visualized using electrochemiluminescence.

### Dual-luciferase reporter assay

The wild-type (WT) or mutant (MUT) circ_0003998 or EIF5A2 3’UTR harboring the potential binding sites of miR-218-5p were amplified and cloned into pmirGLO luciferase reporter vector (Promega, Madison, WI, USA). Then cells were cultured in 24-well plates and co-transfected with wild-type or mutated constructed luciferase reporter plasmid and miR-218-5p or miR-NC using Lipofectamine™ 2000 (Invitrogen). The luciferase activity was detected after 48 h transfection using a dual luciferase assay kit (Promega).

### In vivo chemosensitivity assay

The study was approved by the Animal Research Committee of Shanxi Medical University and manipulated in line with the guidelines of the National Animal Care and Ethics Institution. BALB/c nude mice (male, aged 3–5 weeks, *N* = 12) were divided into four groups of three mice each. Then HCCLM3 (5 × 10^6^ cells) transfected with the lentivirus-(lenti)-sh-circ_0003998 (sh-circ_0003998) or lenti-sh-NC (sh-NC) were subcutaneously injected into the right-side flanks of each mouse, respectively. After the tumor grew to 0.5 mm, two groups transfected with lenti-sh-circ_0003998 or lenti-sh-NC were treated with DOX (3 mg/kg) every 3 days. All nude were fed under Specific Pathogen Free conditions. The volume of the tumor was examined every 4 days using a Vernier caliper. Tumor length and width were measured. Tumors volume was calculated with the equation: Volume = (length × width2)/2. At day 27, the mice were sacrificed by cervical dislocation after deep anesthesia with 2% isoflurane to obtain the tumors, and then tumors were weighed, and stored at − 80°Cfor further molecular analysis.

### Statistical analysis

Data shown are mean values with standard deviation (SD) of at least three experiments. Group comparison was conducted on GraphPad Prism 7 software using one-way analysis of variance (ANOVA) or Student’s *t*-test. The correlation analysis was performed using Pearson correlation analysis. *P* values < 0.05 was considered as statistically significant.

## Results

### Circ_0003998 is elevated in HCC tissues and DOX-resistant HCC cell lines

**Fig. 1:**
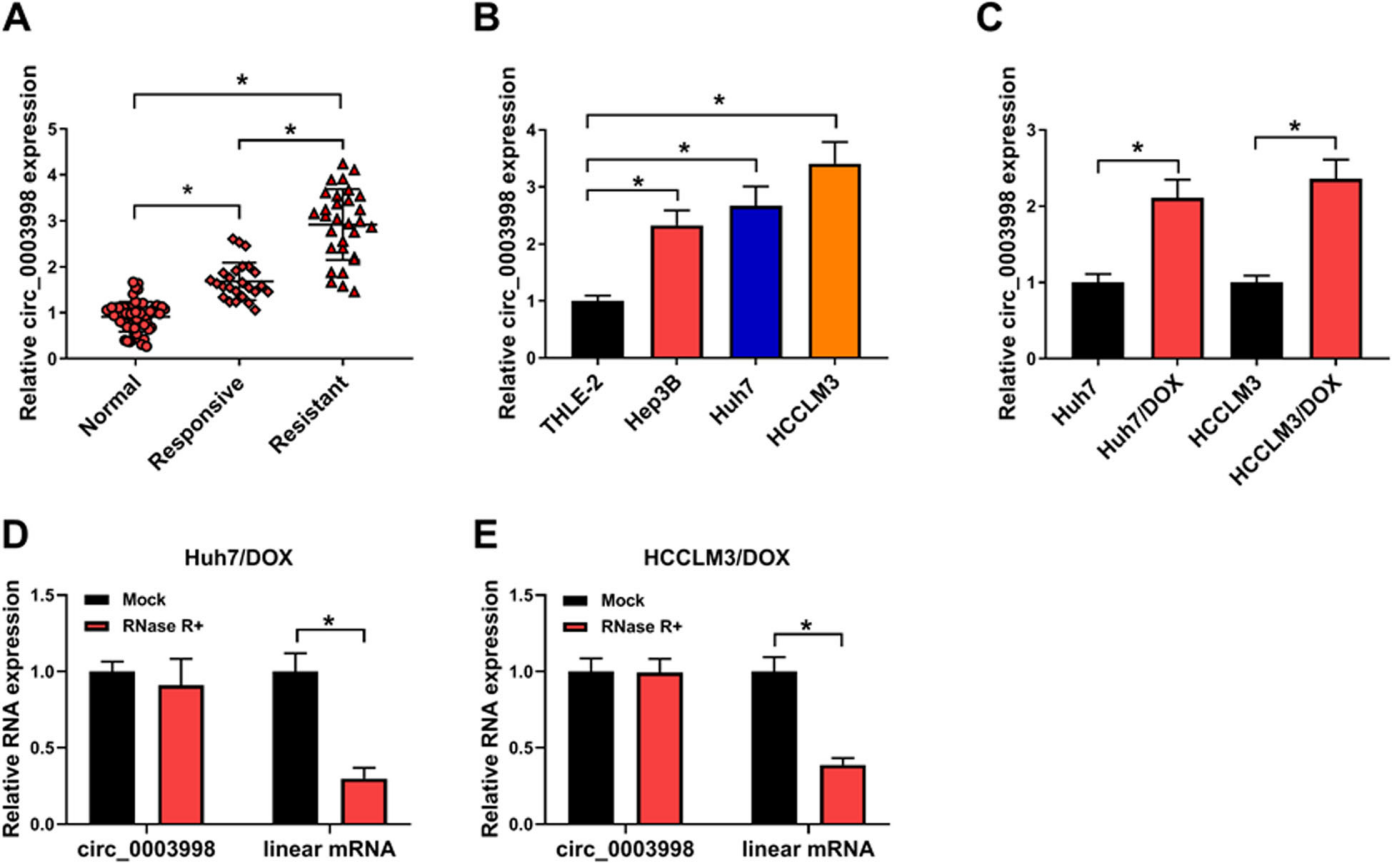
Circ_0003998 is elevated in HCC tissues and DOX-resistant HCC cell lines. **a**-**c** qRT-PCR analysis of circ_0003998 in HCC tissues, DOX-resistant tissues and matched normal tissues (**a**), normal hepatocyte THLE-2 and HCC cell lines (Hep3B, Huh7 and HCCLM3) (**b**), as well as DOX-resistant HCC cell lines (Huh7/DOX and HCCLM3/DOX) and parental HCC cells (Huh7 and HCCLM3) (**c**). **d**, **e** qRT-PCR for the levels circ_0003998 and linear mRNA in Huh7/DOX and HCCLM3/DOX cells after RNase R digestion. *P < 0.05

To explore the impact of circ_0003998 on DOX resistance in HCC cells, the level of circ_0003998 was firstly detected. Results showed circ_0003998 was up-regulated in HCC tissues, especially in DOX-resistant tissues, relative to the matched normal tissues (Fig. 1a). Similarly, qRT-PCR analysis also showed by contrast with normal hepatocyte THLE-2, circ_0003998 was increased in HCC cell lines Hep3B, Huh7 and HCCLM3 (Fig. 1b). Additionally, circ_0003998 expression was elevated in DOX-resistant HCC cell lines (Huh7/DOX and HCCLM3/DOX) compared with the parental HCC cells (Huh7 and HCCLM3) (Fig. 1c). Afterwards, the stability of circ_0003998 in Huh7/DOX and HCCLM3/DOX cells was investigated. Total RNA was digested with or without RNase R and submitted to qRT-PCR reaction, then we found that circ_0003998 was obviously resistant to RNase R compared to the linear mRNA (Fig. 1d, e). These data indicated circ_0003998 was stable and abundant in HCC, and aberrant circ_0003998 overexpression might be associated with DOX resistance in HCC.

### Circ_0003998 knockdown mitigates DOX resistance in HCC cells

**Fig. 2:**
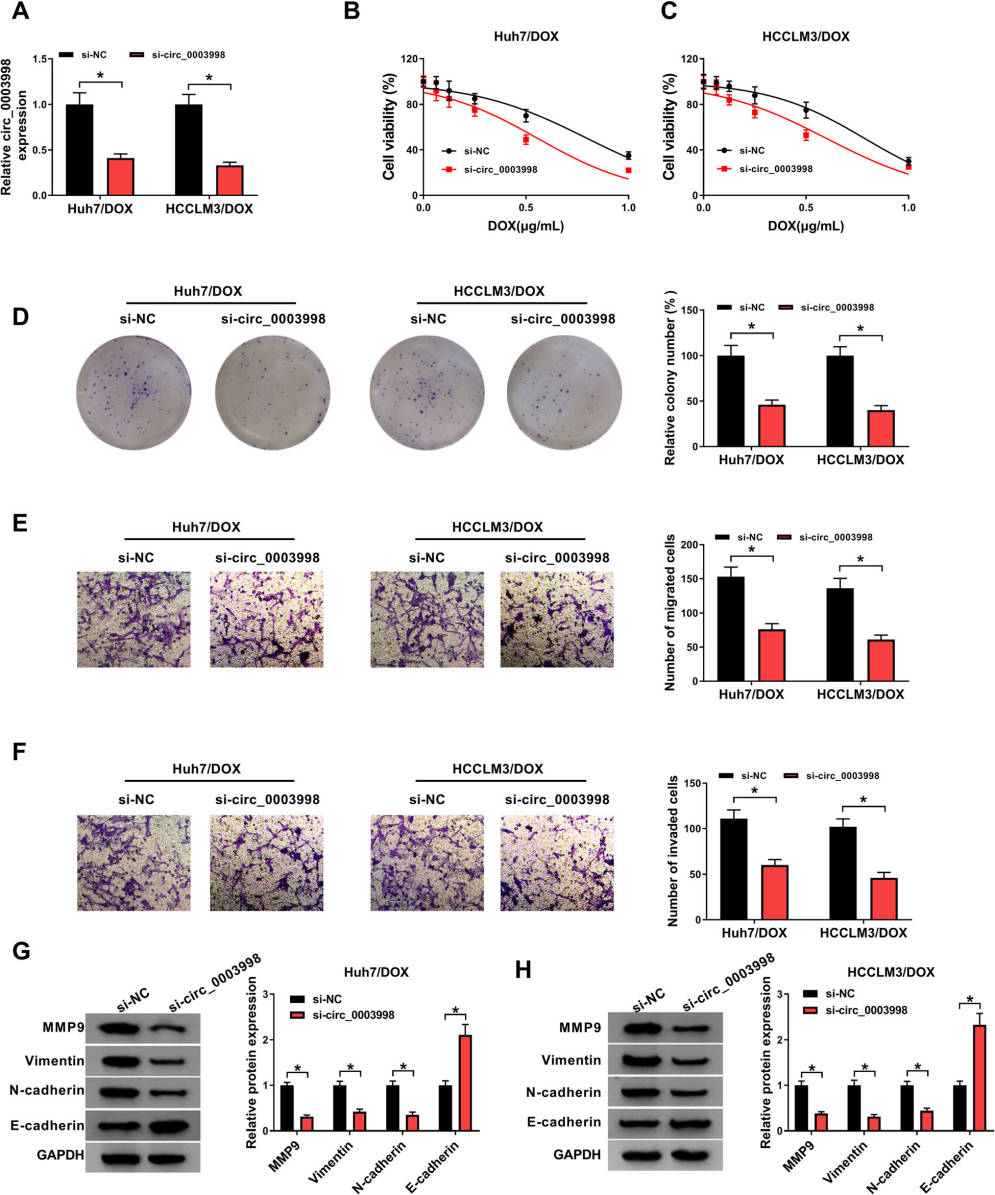
Circ_0003998 knockdown mitigates DOX resistance in HCC cells. Huh7/DOX and HCCLM3/DOX cells were transfected with si-circ_0003998 or si-NC. After transfection, **a** qRT-PCR analysis of circ_0003998 expression; **b**, **c** CCK-8 analysis of resistant cell viability in combination with increasing concentrations of DOX (0, 0.0625, 0.125, 0.25, 0.5, or 1 μg/mL); **d** colony formation analysis of resistant cell viability with 0.5 μg/mL DOX; **e**, **f** transwell analysis of the abilities of migration and invasion in resistant cells; **g**, **h** western blot analysis of MMP9, Vimentin, N-cadherin, and E-cadherin levels in resistant cells. *P < 0.05

Circ_0003998 level was knocked down by transfecting with si-circ_0003998 in DOX-resistant cells to investigate the action of circ_0003998 on DOX resistance. By contrast with si-NC transfection, si-circ_0003998 transfection significantly reduced the expression of circ_0003998 in DOX-resistant cell lines (Fig. 2a). After that, CCK-8 assay indicated circ_0003998 knockdown combined with increasing doses of DOX (0, 0.0625, 0.125, 0.25, 0.5, or 1 μg/mL) gradually inhibited the viability of Huh7/DOX and HCCLM3/DOX cells (Fig. 2b, c). Also, colony formation analysis showed circ_0003998 knockdown combined with 0.5 μg/mL DOX treatment decreased the number of colonies formed (Fig. 2d). After that, transwell assay exhibited the number of migrated and invaded Huh7/DOX and HCCLM3/DOX cells was reduced by circ_0003998 down-regulation (Fig. 2e, f). Additionally, western blot analysis suggested circ_0003998 knockdown decreased the levels of MMP9, Vimentin, N-cadherin, but increased the level of E-cadherin in DOX-resistant cells, indicating circ_0003998 knockdown suppressed the EMT of Huh7/DOX and HCCLM3/DOX cells (Fig. 2g, h). Taken together, circ_0003998 knockdown in DOX-resistant cells suppressed cell viability, migration, invasion, and EMT, sensitizing HCC cells to DOX.

### Circ_0003998 is a sponge of miR-218-5p

**Fig. 3:**
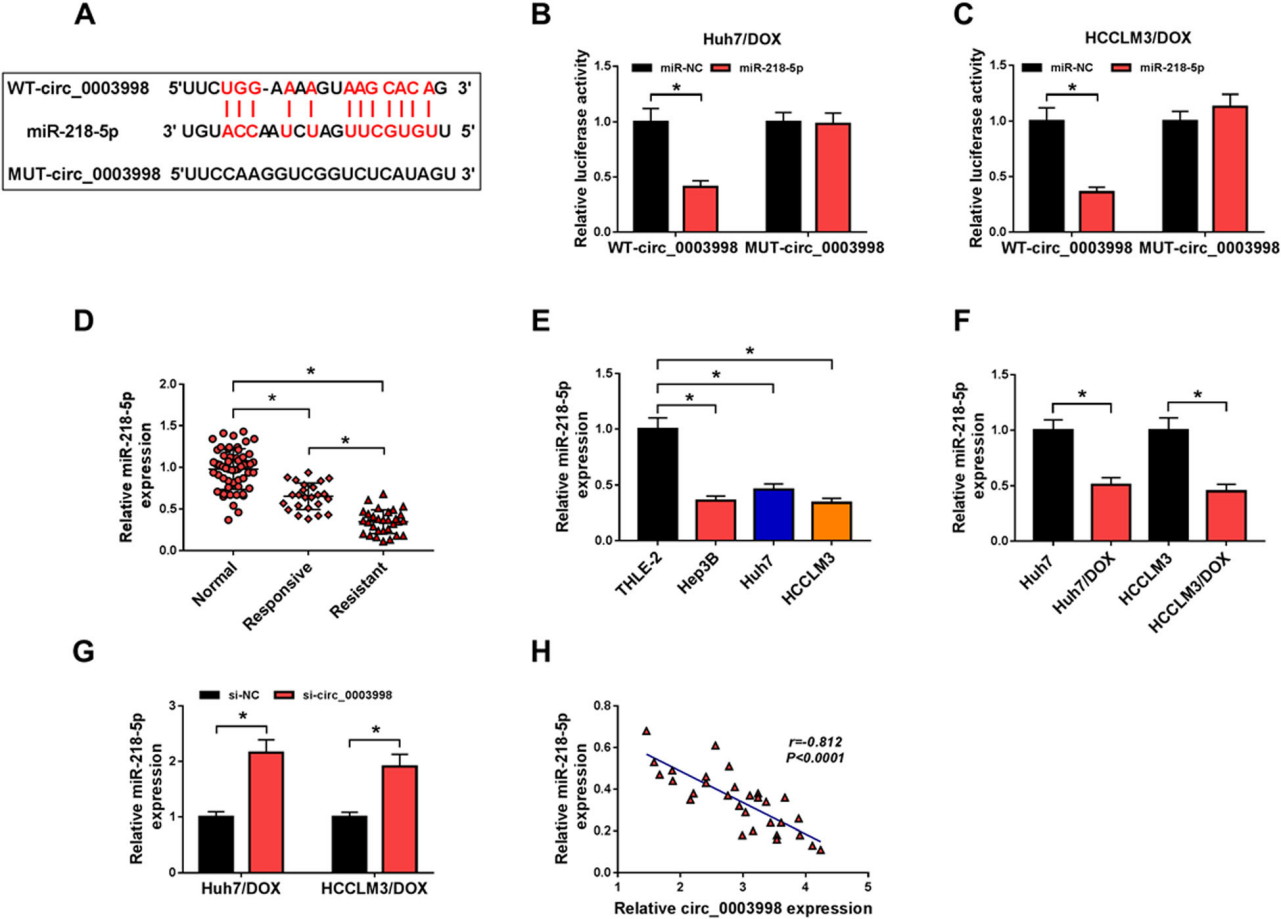
Circ_0003998 is a sponge of miR-218-5p. **a** The potential binding sites of circ_0003998 and miR-218-5p. **b**, **c** Dual-luciferase reporter assay in Huh7/DOX and HCCLM3/DOX cells co-transfected with the reporter plasmid and the indicated miRNAs. **d**-**f** qRT-PCR analysis of miR-218-5p in HCC tissues, DOX-resistant tissues and matched normal tissues (**d**), normal hepatocyte THLE-2 and HCC cell lines (Hep3B, Huh7 and HCCLM3) (**e**), as well as DOX-resistant HCC cell lines and parental HCC cells (**f**). **g** qRT-PCR analysis of miR-218-5p in Huh7/DOX and HCCLM3/DOX transfected with si-NC or si-circ_0003998. **h** Correlation analysis between circ_0003998 and miR-218-5p expression. *P < 0.05

The underlying molecular mechanism of the action of circ_0003998 on DOX resistance was investigated. According to the prediction of Starbase program, the potential binding sits of circ_0003998 and miR-218-5p was listed in Fig. 3a. Immediately, a significantly declined luciferase activity in Huh7/DOX and HCCLM3/DOX cells co-transfected with WT-circ_0003998 and miR-218-5p confirmed the direct interaction between circ_0003998 and miR-218-5p (Fig. 3b, c). MiR-218-5p expression was found to be down-regulated in HCC tissue, especially in DOX-resistant tissues, and cell lines compared to the normal controls (Fig. 3d, e). Furthermore, it also decreased in Huh7/DOX and HCCLM3/DOX cells relative to the parental cells (Fig. 3f), suggesting the potential regulatory roles of miR-218-5p in DOX resistance of HCC. Besides, we also found circ_0003998 knockdown promoted miR-218-5p expression in DOX-resistant cells (Fig. 3g), and miR-218-5p expression was negatively correlated with circ_0003998 (Fig. 3h). These data verified that circ_0003998 was a sponge of miR-218-5p and negatively regulated its expression.

### Circ_0003998 knockdown sensitizes HCC cell to DOX by sponging miR-218-5p

**Fig. 4:**
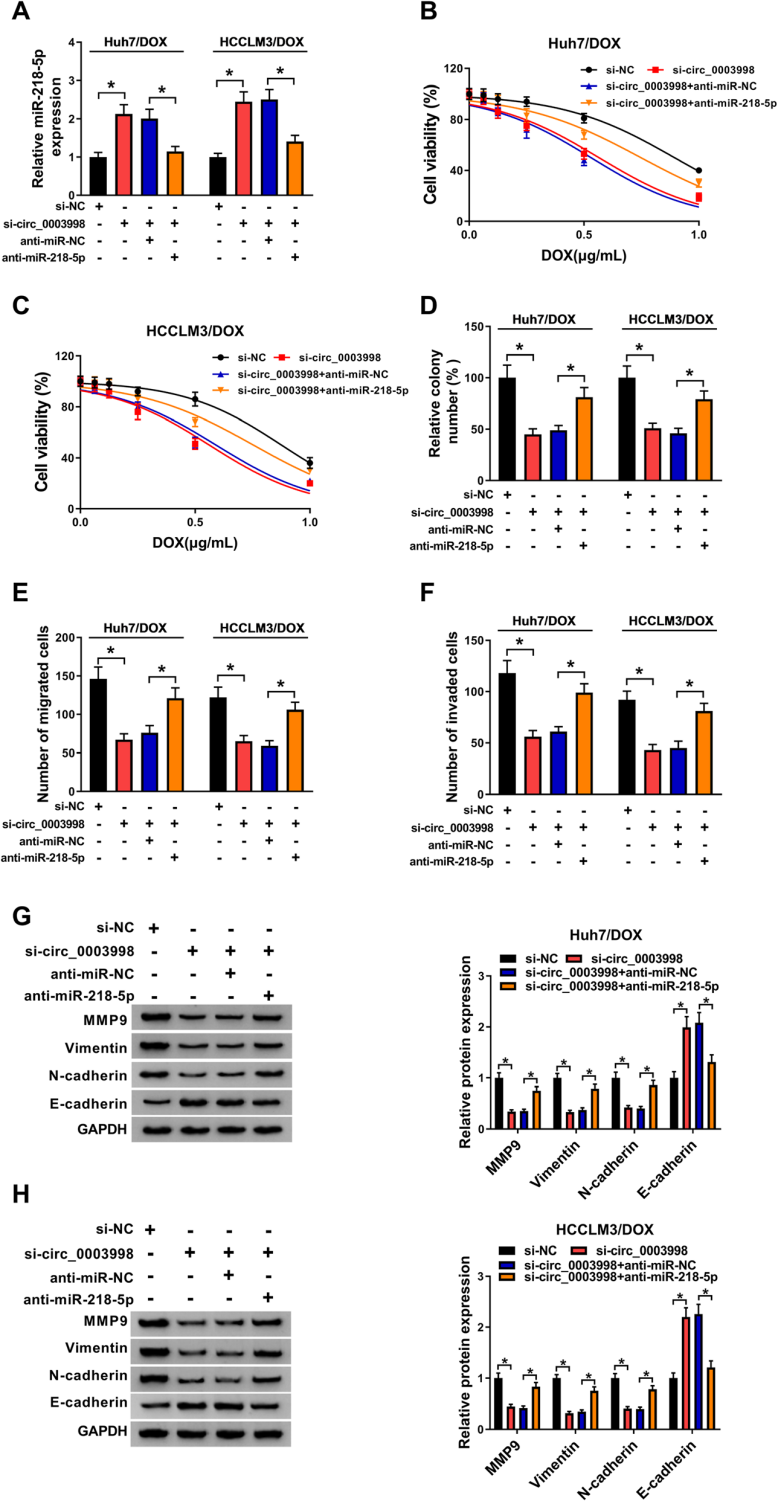
Circ_0003998 knockdown sensitizes HCC cell to DOX by sponging miR-218-5p. Huh7/DOX and HCCLM3/DOX cells were transfected with si-NC, si-circ_0003998, si-circ_0003998 + anti-miR-NC, or si-circ_0003998 + anti-miR-218-5p. After transfection, **a** qRT-PCR analysis of miR-218-5p expression; **b**-**d** the viability analysis of resistant cell in combination with DOX using CCK-8 assay and colony formation analysis; **e**, **f** migration and invasion analysis of resistant cells with transwell assay; **g**, **h** levels analysis of MMP9, Vimentin, N-cadherin, and E-cadherin in resistant cells using western blot. *P < 0.05

We further studied whether miR-218-5p involved in the action of circ_0003998 on HCC cell DOX resistance, Huh7/DOX and HCCLM3/DOX cells were transfected with si-NC, si-circ_0003998, si-circ_0003998 + anti-miR-NC, or si-circ_0003998 + anti-miR-218-5p, and qRT-PCR analysis showed si-circ_0003998 transfection notably promoted miR-218-5p expression, but this promotion was attenuated by miR-218-5p inhibition (Fig. 4a), validating the successful transfection. Then CCK-8 and colony formation assays showed that knockdown of circ_0003998 significantly decreased the DOX resistance of Huh7/DOX and HCCLM3/DOX cells, while miR-218-5p inhibition rescued the effects (Fig. 4b-d). Besides that, miR-218-5p inhibition also reversed circ_0003998 silence-induced suppression on Huh7/DOX and HCCLM3/DOX cell migration (Fig. 4e), invasion (Fig. 4f) and EMT (Fig. 4g, h). Altogether, circ_0003998 silence promoted DOX-sensitivity in HCC cells by regulating miR-218-5p.

### EIF5A2 is a target of miR-218-5p

**Fig. 5:**
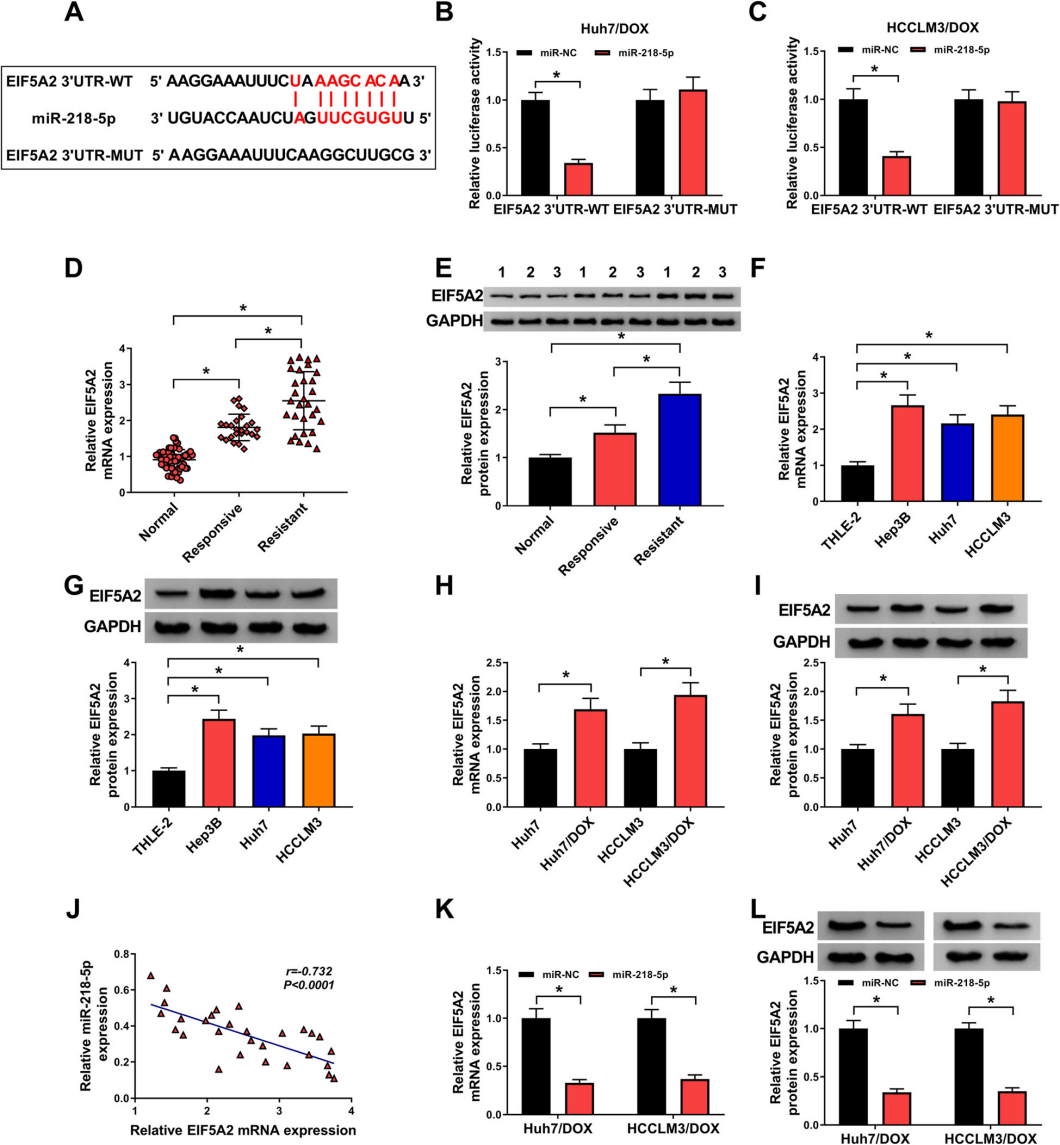
EIF5A2 is a target of miR-218-5p. **a** The binding sites of miR-218-5p on EIF5A2. **b**, **c** Dual-luciferase reporter assay in Huh7/DOX and HCCLM3/DOX cells co-transfected with the reporter plasmid and the indicated miRNAs. **d**-**i** mRNA and protein levels analysis of EIF5A2 in HCC tissues, DOX-resistant tissues and matched normal tissues (**d**, **e**), normal hepatocyte THLE-2 and HCC cell lines (Hep3B, Huh7 and HCCLM3) (**f**, **g**), as well as DOX-resistant HCC cell lines and parental HCC cells (**h**, **i**) using qRT-PCR and western blot. **j** Correlation analysis between EIF5A2 and miR-218-5p expression. **k**, **l** Levels analysis of EIF5A2 expression in Huh7/DOX and HCCLM3/DOX cells transfected with miR-NC or miR-218-5p using qRT-PCR and western blot. *P < 0.05

By searching the starbase program, the binding sites of miR-218-5p on EIF5A2 was observed (Fig. 5a). Then a dual luciferase reporter assay displayed that miR-218-5p overexpression significantly reduced the luciferase activity of the EIF5A2 3’UTR-WT reporter vector but not mutant reporter vector in Huh7/DOX and HCCLM3/DOX cells (Fig. 5b, c). EIF5A2 expression was up-regulated in HCC tissues, especially in DOX-resistant tissues, (Fig. 5d, e) and cell lines (Fig. 5f, g) at mRNA and protein levels, and also elevated in Huh7/DOX and HCCLM3/DOX cells compared with the parental cells (Fig. 5h, i). Besides that, EIF5A2 expression was negatively correlated with miR-218-5p (Fig. 5j) and was repressed by miR-218-5p overexpression (Fig. 5k, l). In all, miR-218-5p targetedly repressed EIF5A2 expression.

### MiR-218-5p alleviates DOX resistance in HCC cells through modulating EIF5A2 expression

**Fig. 6:**
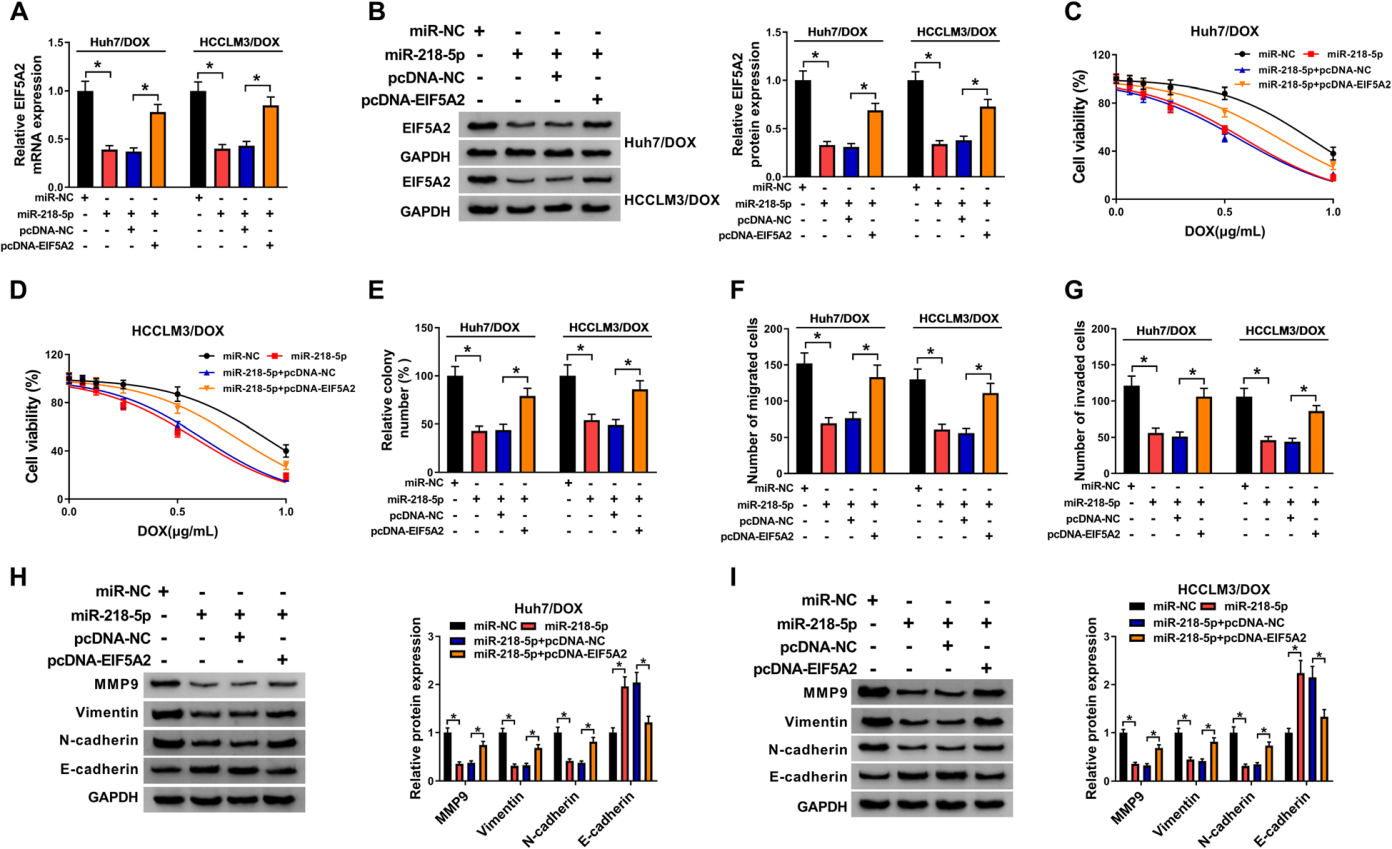
MiR-218-5p alleviates DOX resistance in HCC cells through modulating EIF5A2 expression. Huh7/DOX and HCCLM3/DOX cells were transfected with miR-NC, miR-218-5p, miR-218-5p + pcDNA-NC, or miR-218-5p + pcDNA-EIF5A2. After transfection, **a**, **b** qRT-PCR and western blot analysis of EIF5A2 levels in resistant cells. **c**, **d** CCK-8 analysis of resistant cell viability in combination with increasing concentrations of DOX (0, 0.0625, 0.125, 0.25, 0.5, or 1 μg/mL); **e** colony formation analysis of resistant cell viability with 0.5 μg/mL DOX; **f**, **g** transwell analysis of the abilities of migration and invasion in resistant cells; **h**, **i** level analysis of MMP9, Vimentin, N-cadherin, and E-cadherin in resistant cells with western blot. *P < 0.05

We then evaluate whether miR-218-5p/EIF5A2 axis regulated DOX resistance in HCC. Huh7/DOX and HCCLM3/DOX cells were transfected with miR-NC, miR-218-5p, miR-218-5p + pcDNA-NC, or miR-218-5p + pcDNA-EIF5A2, and we found EIF5A2 up-regulation rescued miR-218-5p restoration-mediated inhibition on EIF5A2 expression (Fig. 6a, b), suggesting the successful transfection. After that, CCK-8 and colony formation assays displayed that miR-218-5p combined with DOX notably decreased the viability of Huh7/DOX and HCCLM3/DOX cells, while EIF5A2 up-regulation reversed this effect (Fig. 6c-e). In addition, transwell assay exhibited that EIF5A2 up-regulation abated miR-218-5p overexpression-induced repression of Huh7/DOX and HCCLM3/DOX cell migration and invasion (Fig. 6f, g). Importantly, western blot suggested miR-218-5p blocked DOX resistance in Huh7/DOX and HCCLM3/DOX cells via suppressing cell EMT, but this effect was neutralized by EIF5A2 up-regulation (Fig. 6h, i). These results indicated miR-218-5p suppressed DOX resistance in HCC cells by targeting EIF5A2.

### Circ_0003998 serves as a competing endogenous RNA (ceRNA) for miR-218-5p to regulate EIF5A2 expression

**Fig. 7:**
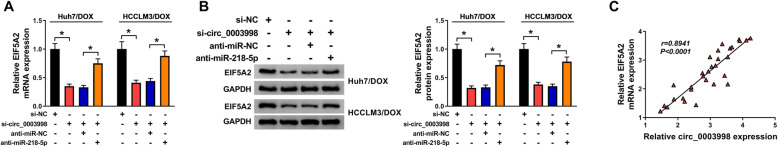
Circ_0003998 serves as a ceRNA for miR-218-5p to regulate EIF5A2 expression. **a**, **b** Western blot analysis of EIF5A2 expression in Huh7/DOX and HCCLM3/DOX cells transfected with si-NC, si-circ_0003998, si-circ_0003998 + anti-miR-NC, or si-circ_0003998 + anti-miR-218-5p. **c** Correlation analysis between EIF5A2 and circ_0003998 expression. *P < 0.05

Whether circ_0003998 regulated DOX resistance via the miR-218-5p/EIF5A2 axis was further determined. Western blot analysis showed circ_0003998 knockdown negatively affected EIF5A2 level in Huh7/DOX and HCCLM3/DOX cells, while this down-regulation of EIF5A2 expression was mitigated by following miR-218-5p inhibition (Fig. 7a, b); besides, a positively correlation between EIF5A2 and circ_0003998 was also investigated (Fig. 7c). Thus, we concluded that circ_0003998 could positively regulated EIF5A2 by binding to miR-218-5p.

### Circ_0003998 silence enhances the cytotoxicity of DOX on HCC cells in vivo

**Fig. 8:**
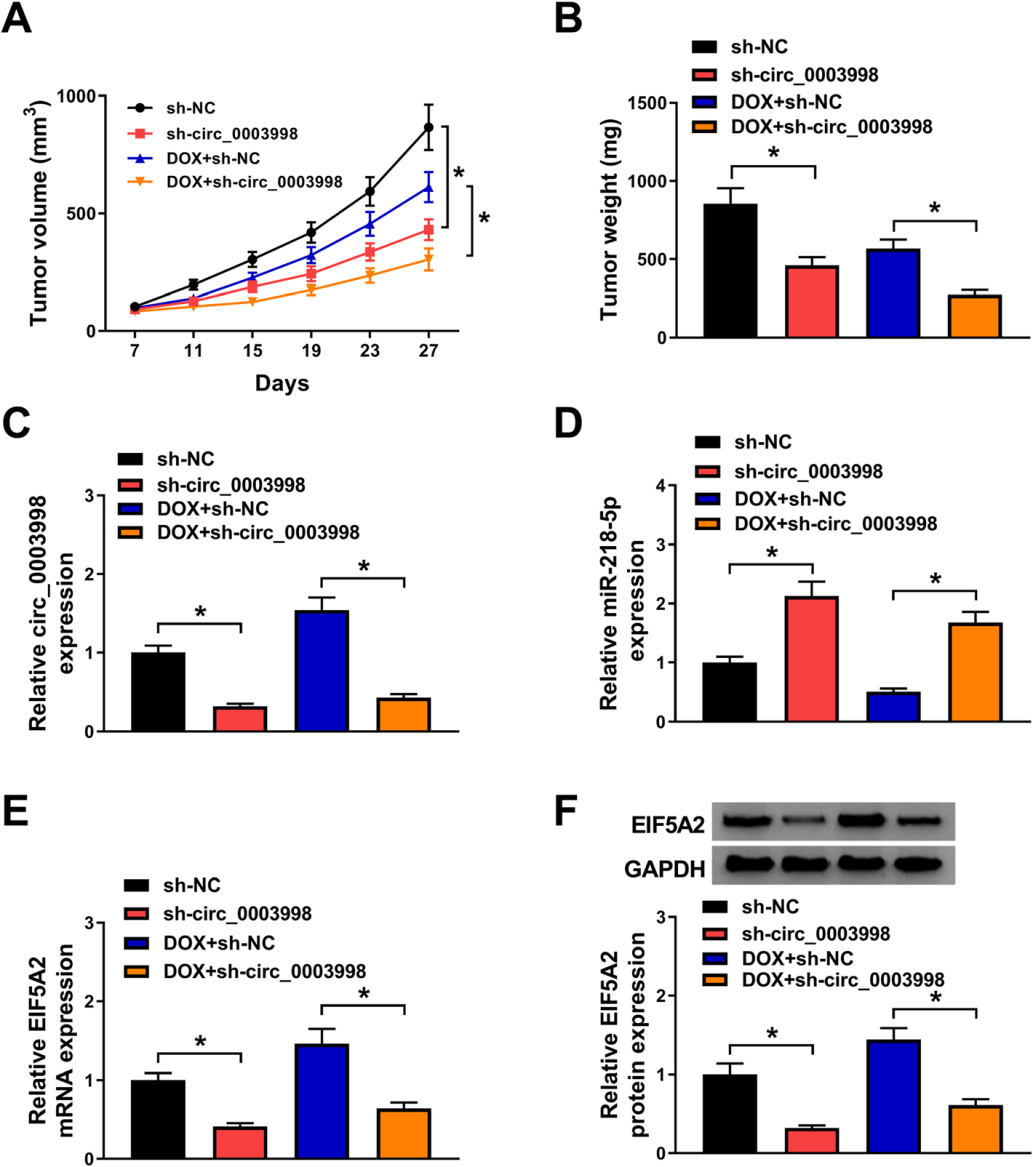
Circ_0003998 silence enhances the cytotoxicity of DOX on HCC cells in vivo. **a** Tumor volumes were calculated every 4 days. **b** Mice were sacrifice on day 27 and tumor masses were excised and weighed. **c**-**e** The expression of circ_0003998, miR-218-5p and EIF5A2 mRNA in the tumor tissues in each group quantified using qRT-PCR. **f** Protein expression of EIF5A2 in the tumor tissues in each group quantified using western blot. *P < 0.05

The effects of circ_0003998 on DOX-mediated tumor growth in vivo was verified. The results suggested circ_0003998 knockdown inhibited HCC tumor growth; more importantly, the circ_0003998 knockdown significantly enhanced DOX-mediated suppression of tumor growth in vivo, reflected by tumor growth curve and tumor weights (Fig. 8a, b). In addition, molecular analysis showed the injection of sh-circ_0003998 was successful and the circ_0003998 levels were decreased in the tumor masses (Fig. 8c). In the meanwhile, the level of miR-218-5p and EIF5A2 in the tumor tissues was determined and results indicated that circ_0003998 knockdown elevated miR-218-5p and reduced EIF5A2 expression in vivo (Fig. 8d-f). Therefore, circ_0003998 knockdown enhanced DOX-mediated repression of HCC tumor growth by regulating miR-218-5p/EIF5A2 in vivo.

## Discussion

HCC is an aggressive malignancy with the high recurrence rate. The clinical therapeutic effects of conventional methods are always unsatisfactory due to the development of chemoresistance [15, 16]. Up to date, a growing body of studies have demonstrated circRNAs contributed to the tumorigenesis of HCC, simultaneously, circRNAs modulate the chemoresistance of HCC cells [17–19]. Therefore, circRNAs might be promising targets for chemoresistance of DOX by regulating tumor cell biological behavior. In this study, circ_0003998 was observed to be stable and abundant in HCC tissues and cells, particularly in DOX-resistant tissues and cells, which suggested that high circ_0003998 expression had association with DOX resistance. Then we found that silencing of circ_0003998 sensitized HCC cell to DOX by inhibiting resistant cell viability, migration, invasion and EMT. In addition, circ_0003998 knockdown also enhanced DOX cytotoxicity in HCC cells, thus repressing tumor growth in vivo*.*

MicroRNAs (miRNA) are small non-protein coding RNA molecules which control gene expression at post-transcriptional level [20]. Growing evidence has documented that miRNAs involve in the regulation of all aspects of cancer biology, including cell proliferation, migration, apoptosis, EMT and drug resistance [21, 22]. Importantly, the up-regulation or down-regulation of some miRNAs had been implied to ameliorate or enhance drug resistance in HCC [23]. MiR-218-5p is a well-recognized tumor suppressor in a variety of tumors. For example, miR-218-5p suppressed cell proliferation and migration in NSCLC by interacting with EGFR [24]. MiR-218-5p restoration restrained the deterioration of glioma by suppressing cancer cell proliferation, migration, and EMT [25]. In HCC, miR-218-5p also was found to function as a tumor suppressor to inhibit the progression of HCC [26, 27]. In this study, miR-218-5p was decreased in DOX-resistant cells compared with the parental cells, and miR-218-5p restoration inhibited DOX resistance in HCC cells. Importantly, we verified miR-218-5p was a target of circ_0003998, and was negatively regulated by circ_0003998, and circ_0003998 knockdown sensitized HCC cell to DOX by binding to miR-218-5p.

EIF5A is a translation factor which influences both initiation and elongation and involves in transcription, mRNA turnover, and nucleocytoplasmic transport; besides that, EIF5A has also found to implicate in oncogenic activities [28]. EIF5A2, a second isoform of EIF5A, has been observed to be up-regulated in many type tumors and is an important carcinogenic biomarker in many cancers [28, 29]. Also, previous studies probed that EIF5A2 was involved in the progression of HCC by regulating tumor cell metabolic reprogramming, migration, invasion, proliferation, and oxidative stress [30–32]. Additionally, abnormal EIF5A2 also promoted the cetuximab [33, 34] and DOX [35] resistance by regulating tumorigenic properties in HCC. In this review, EIF5A2 was elevated in DOX-resistant cells. We then confirmed the direct interaction of miR-218-5p and EIF5A2; besides that, rescue assay showed miR-218-5p alleviated DOX resistance in HCC cells through modulating EIF5A2 expression. Moreover, we also observed that circ_0003998 functioned as a ceRNA of miR-218-5p to modulate EIF5A2 expression.

## Conclusion

In conclusion, this study demonstrated that circ_0003998 knockdown sensitized HCC cell to DOX by inhibiting tumorigenic properties and tumor growth of resistant cells through miR-218-5p/EIF5A2 axis, indicating a potential molecular targeted therapy strategy for the intervention of HCC chemoresistance.

## Supplementary Information

**Additional file 1.** The CT curves of circ_0003998, miR-218-5p and EIF5A2 mRNA.

## Data Availability

The analyzed data sets generated during the present study are available from the corresponding author on reasonable request.

## Abbreviations

circRNAs: Circular RNAs
DOX: Doxorubicin
HCC: Hepatocellular carcinoma
EIF5A2: Eukaryotic translation initiation factor 5A-2
MMP-9: Metallopeptidase 9
TACE: Transarterial chemoembolization
NSCLC: Non-small cell lung cancer
qRT-PCR: Quantitative real-time polymerase chain reaction
DMEM: Dulbecco’s modified Eagle’s medium
FBS: Fetal bovine serum
