# Supplementary material for: Circ_0003998 enhances doxorubicin resistance in hepatocellular carcinoma by regulating miR-218-5p/EIF5A2 pathway
Source: Diagn Pathol. 2020 Dec 11;15:141. doi: 10.1186/s13000-020-01056-1 (PMC7733254; doi:10.1186/s13000-020-01056-1)
Supplement: Supplementary file 1 — Additional file 1. The CT curves of circ_0003998, miR-218-5p and EIF5A2 mRNA. [file 13000_2020_1056_MOESM1_ESM.pdf]

GAPDH:

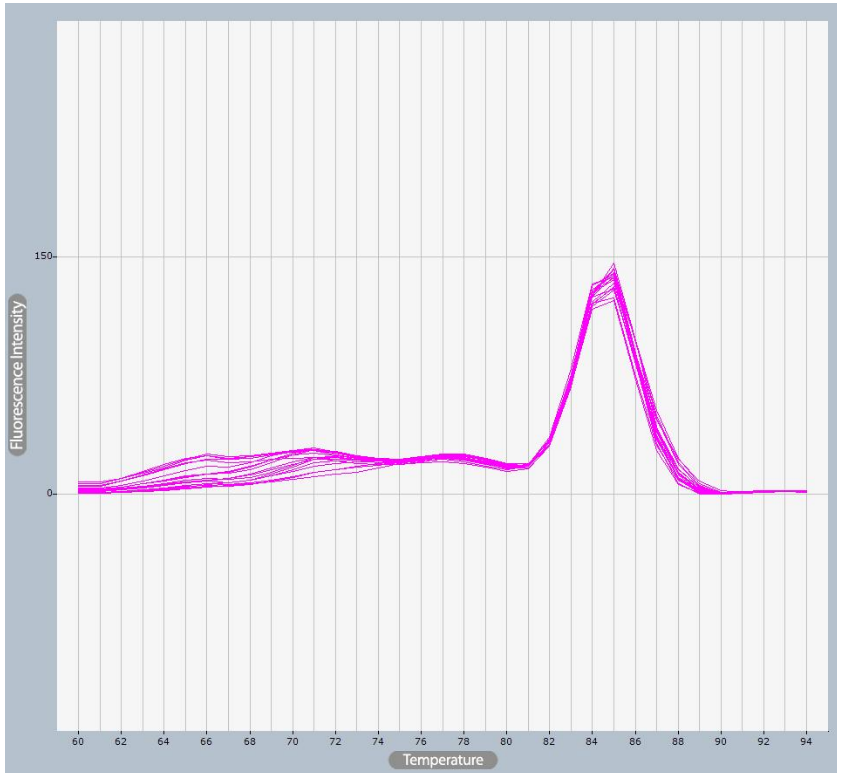

GAPDH melting

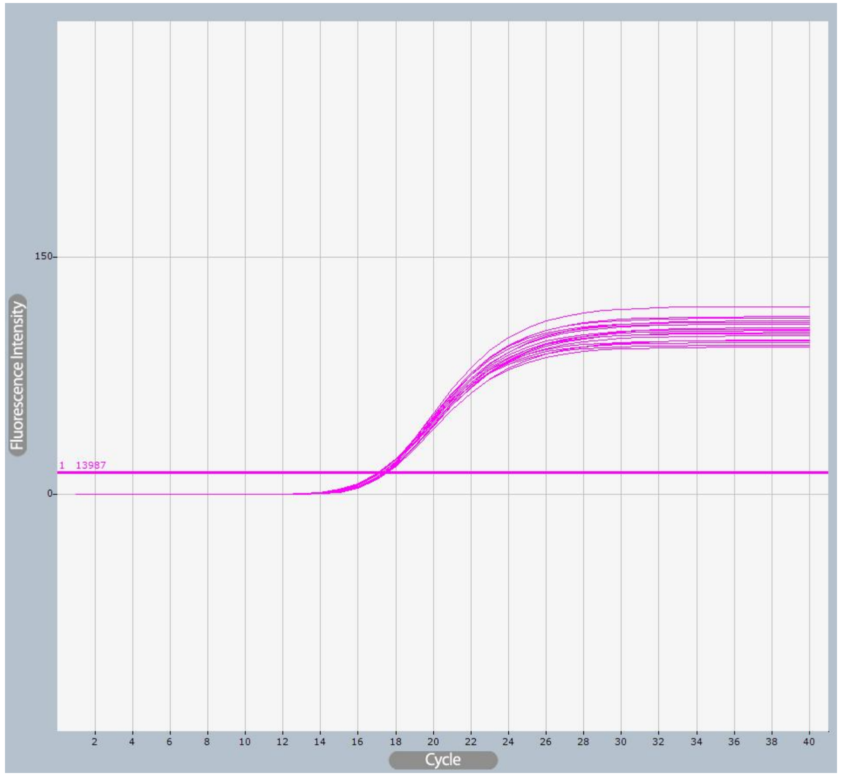

GAPDH

U6

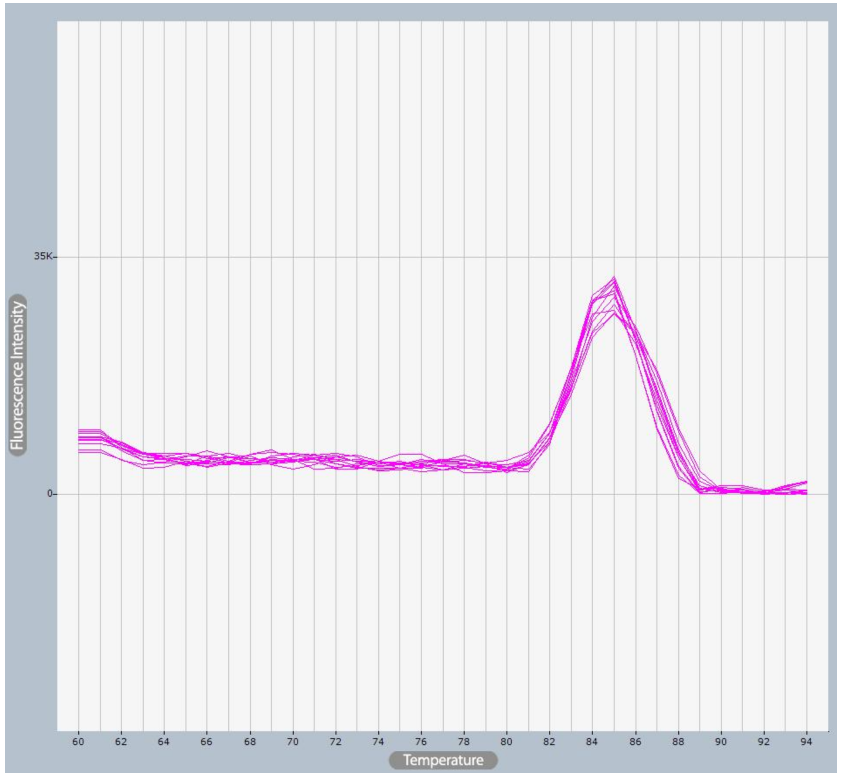

U6 melting

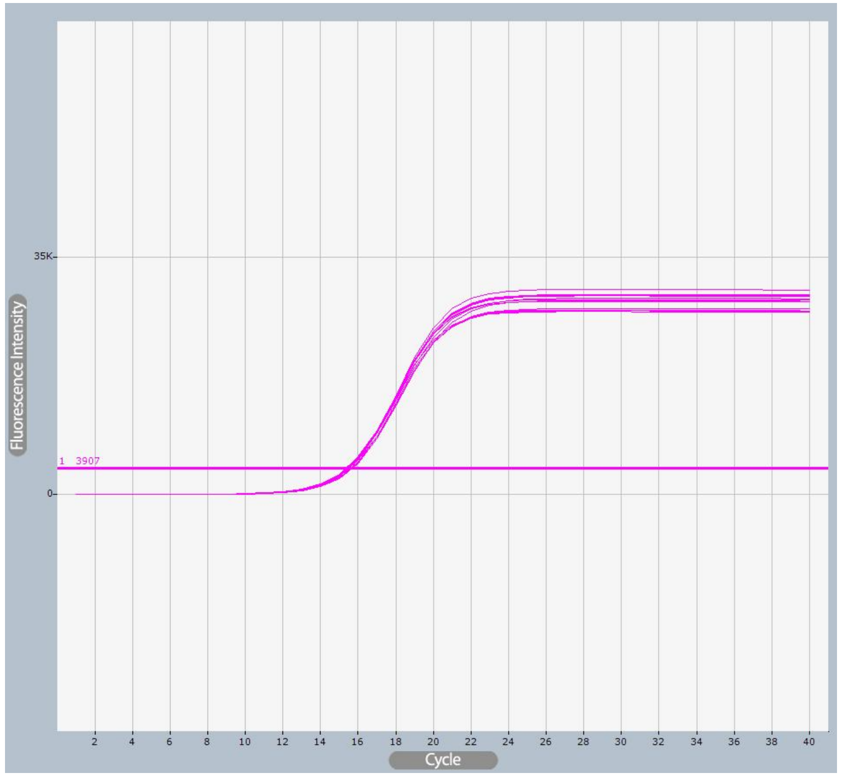

U6

hsa_circ_0003998

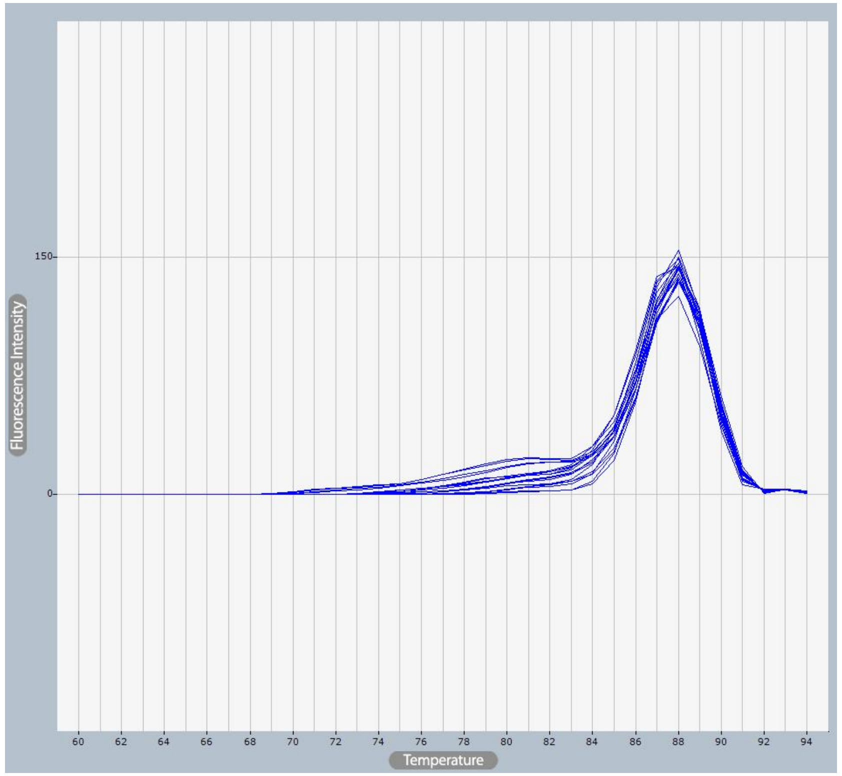

circ_0003998 melting

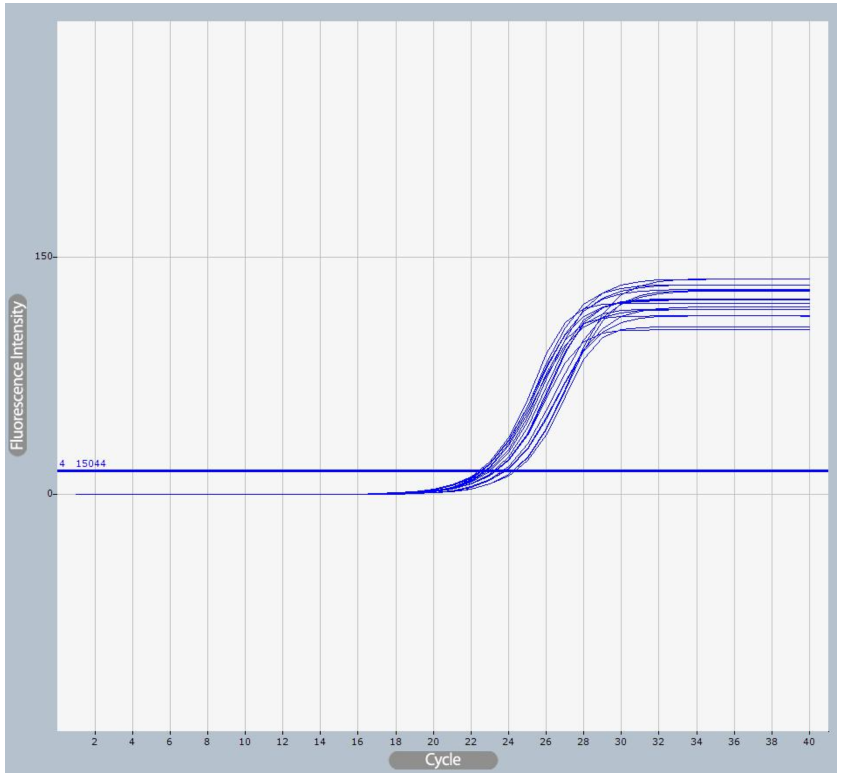

circ_0003998

miR-218-5p

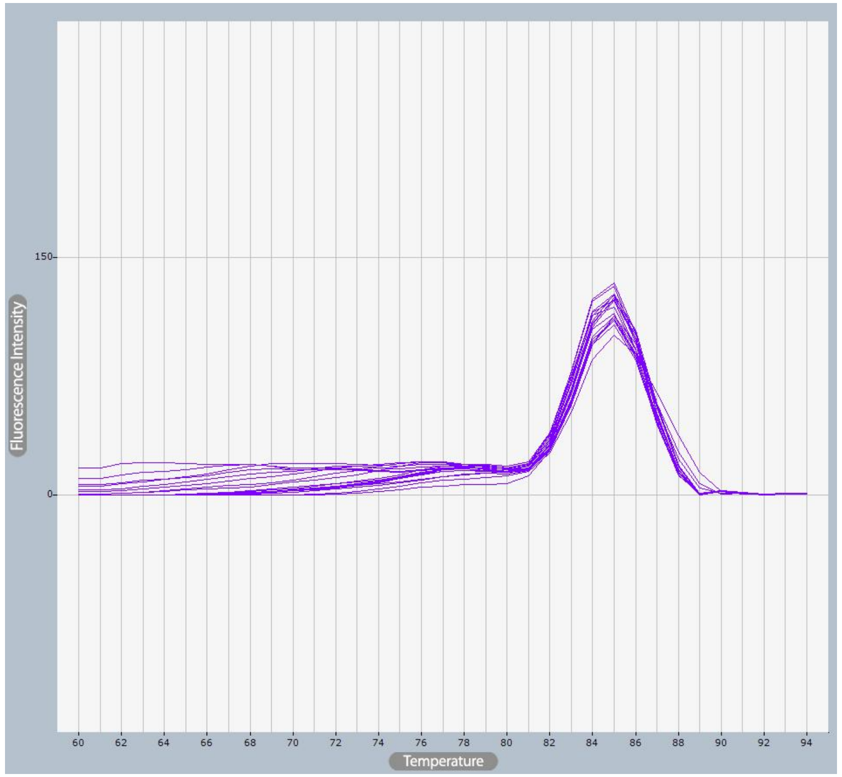

miR-218-5p melting

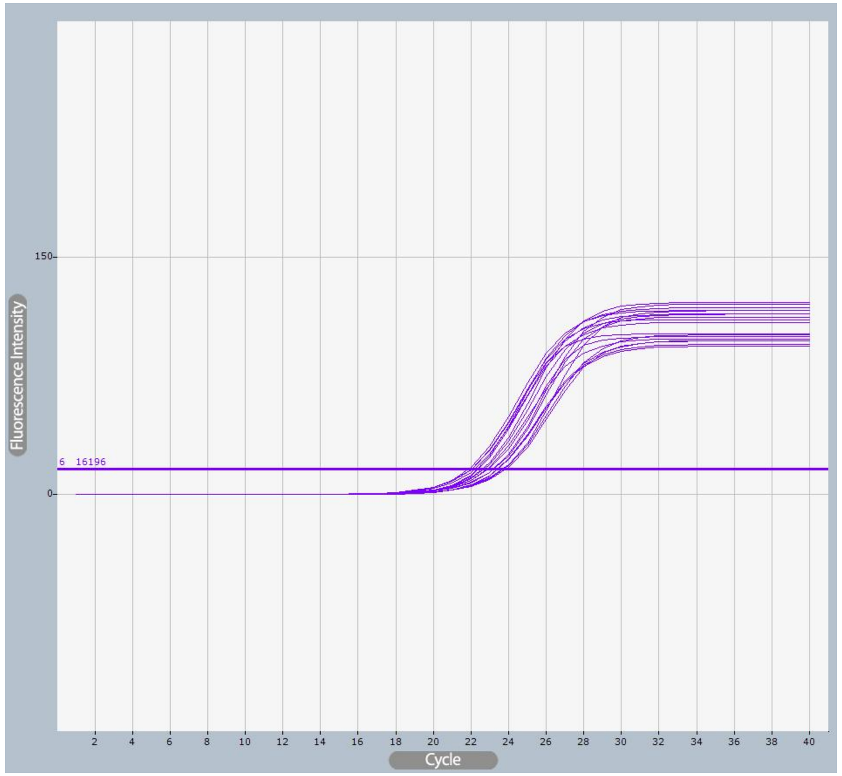

miR-218-5p

EIF5A2

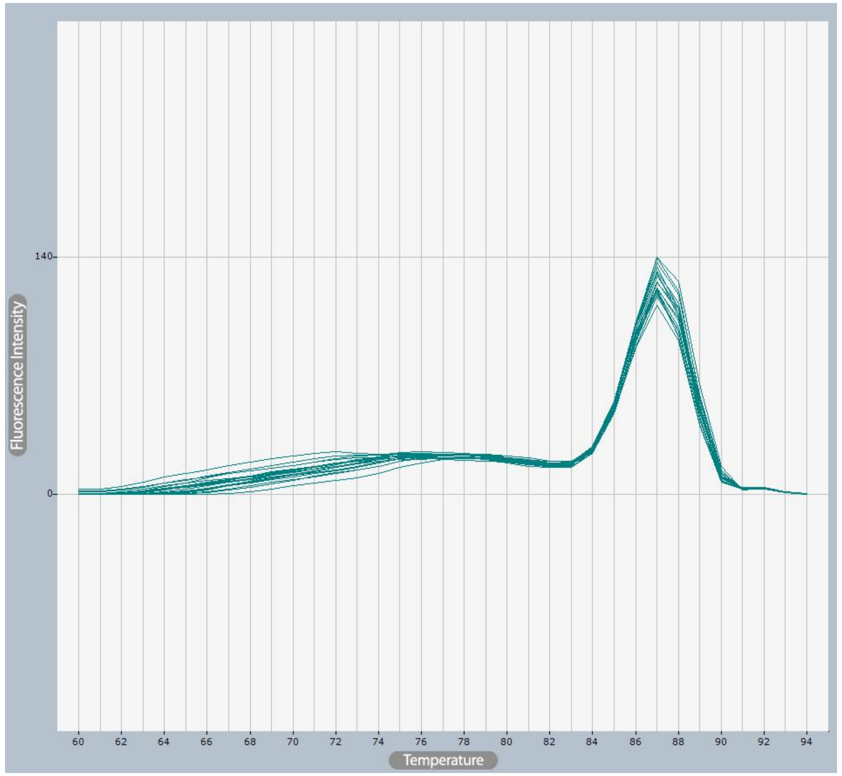

EIF5A2 melting

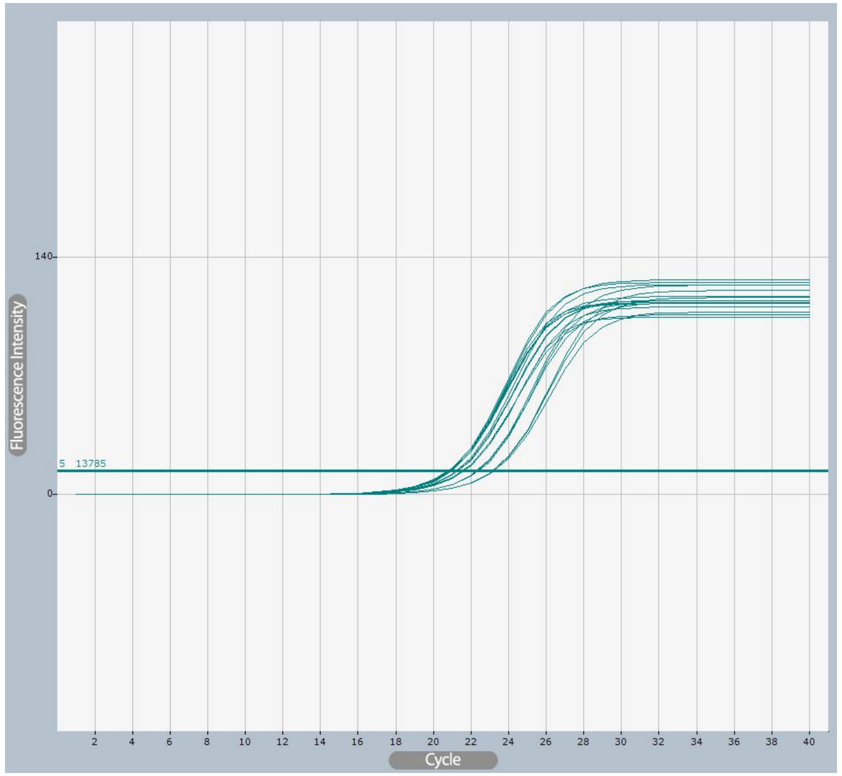

EIF5A2
